# A humanistic model of corporate social responsibility in e-commerce with high-tech support in the artificial intelligence economy

**DOI:** 10.1057/s41599-023-01764-1

**Published:** 2023-05-31

**Authors:** Elena B. Zavyalova, Vera A. Volokhina, Marija A. Troyanskaya, Yulia I. Dubova

**Affiliations:** 1 Moscow State Institute of International Relations (MGIMO University), Moscow, Russia; 2 Orenburg State University, Orenburg, Russia; 3 Volgograd State Technical University, Volgograd, Russia

**Keywords:** Business and management, Economics, Information systems and information technology

## Abstract

This paper aims to develop a humanistic model of corporate social responsibility in e-commerce, relying on high technology in an artificial intelligence economy. The research is based on the experience of the top 30 publicly traded e-commerce companies, the 16 most responsible companies in the retail industry in the USA, and the leading global and Russian e-commerce business structures in 2020–2021. Based on econometric modeling, it is substantiated that the humanization (qualitative criterion) of jobs provides an increase in revenues of e-commerce businesses to a greater extent than an increase in the number (quantitative criterion) of jobs. The high technology of the artificial intelligence economy (AI economy) makes it possible to maximize the contribution of responsible HRM of the e-commerce business in increasing its revenues. For this purpose, a humanistic model of corporate social responsibility in e-commerce based on high technology in the AI economy has been developed. The theoretical significance lies in proving the need to humanize jobs in e-commerce and revealing the essence of this process. The practical significance lies in the fact that the developed humanistic model will increase the profitability and, consequently, the resilience of businesses to future economic crises that arise against the backdrop of the COVID-19 pandemic.

## Introduction

The relevance of studying the prospects for the humanization of e-commerce is due to the fact that in the AI economy, the role of this new sphere is very large, and it has particularly grown in recent years. With the pandemic and the COVID-19 crisis, there was a drop in business revenues (Untaru and Han, 2021). On the one hand, this is due to economic factors such as inflation and increased production costs due to the disruption of global supply and sales chains and the need to transform them, which, in many cases, involves entering into contracts on less favorable terms. On the other hand, the reasons include social factors, among which the main ones are the limitation of sales opportunities due to the self-isolation of consumers, the reduction of labor productivity, and the degree of utilization of the production capacity of the business due to employee illness, the introduction of social distancing measures in the business processes, and the transfer of employees to remote work.

The mechanism for reducing business revenues in the COVID-19 pandemic and crisis is as follows. When a lockdown is imposed under the combined effect of the social and economic factors noted, there is a decrease in production and a reduction in effective demand. Against a general macroeconomic downturn, society places greater demands on the corporate social responsibility of business (Santos et al., 2022). Multiple studies, for example, studies by Asokan et al. (2022), El Khoury et al. (2022), and Miller et al. (2022) show that consumers are favoring responsible business products over irresponsible business products during the COVID-19 pandemic and crisis. This points to the close connection between business revenues and the level of its corporate social responsibility in the COVID-19 pandemic and crisis.

The problem is that while the existence of this connection has been scientifically proven, its essence remains unclear. Bianchet et al. (2021) and Jasielska et al. (2022) note an increase in consumer loyalty and provide evidence of increased demand for responsible business products. This means improving the external (driven by exogenous reasons from consumers) quantitative characteristics of business performance. However, it seems that the available explanation of the essence of the link between business income and the level of corporate social responsibility during the COVID-19 pandemic and crisis is incomplete because it does not consider the role of business employees.

Responsible human resource management (HRM) largely determines actual and perceived by external stakeholders (including consumers) corporate social responsibility of business. In particular, consumers recognize and appreciate the corporate social responsibility of business, seeing it not only as a reliable supplier of socially important products but also as a responsible employer. Ankiewicz (2021), He et al. (2021), and Lim et al. (2021) point to the important place of responsible HRM in linking business revenues to its level of corporate social responsibility in the COVID-19 pandemic and crisis. Nevertheless, these authors leave the role of responsible HRM in this process unclear. This is a gap in the literature that this research seeks to fill.

The research question (RQ) is as follows: “How does responsible HRM contribute to business revenues in the COVID-19 pandemic and crisis, and how can we maximize this contribution?” The significance of filling the identified gap in the literature and finding an answer to the RQ is due to the fact that, despite initial expectations of a quick end to the COVID-19 pandemic, it is now in its third year, and there are no signs of an imminent end. Although the COVID-19 economic crisis ended in 2020, more outbreaks of the new coronavirus infection are occurring despite mass vaccination. The risk of exacerbation of the COVID-19 disease situation remains. Strict sanitary and epidemiological restrictions and quarantine measures, if required and imposed, could cause a new wave of economic crisis.

The need to fill the discovered gap in the literature and to search for an answer to the posed RQ is explained by the fact that it is important to study the accumulated experience and clarify the causal links between the corporate social responsibility of business and its profits in the economic crisis of a pandemic nature in the context of the ongoing COVID-19 pandemic, in the post-pandemic period, as well as in the context of increasing business resilience to future economic crises arising from pandemics.

Separate attention should be paid to the experience of e-commerce, which optimally meets the requirements for social distancing because it mainly involves remote employment and remote business communication with consumers. Due to this, e-commerce became particularly popular under the conditions of the COVID-19 lockdown.

An increase in the development of e-commerce during the most acute period of the pandemic (2020) was predetermined by the fact that it allowed retaining the normal work of business and preserving its presence in the market, while the offline form of its activities was impossible because of the large number of sick employees and because of the imposed coronavirus restrictions, as well as due to responsible consumers’ concerns about their health and their voluntary refusal from offline purchases in favor of e-commerce.

E-commerce was made possible by the emergence of an artificial intelligence economy. However, the impact of high technology on responsible HRM is controversial and needs to be clarified, given the special conditions of the COVID-19 pandemic. For example, automation can reduce the staffing needs of e-commerce businesses, causing downsizing and unemployment. Simultaneously, working conditions optimally meet epidemiological standards for the remaining workers whose jobs have been preserved.

Recognizing the relevance of the problem posed, this research seeks to develop a humanistic model of corporate social responsibility in e-commerce, relying on high technology in an AI economy. The stated goal determined the order and design of this research. The authors conduct a literature review, which results in a gap analysis and hypothesis. Next, they describe the materials and methodology.

In the results, the authors perform the following:

1. Assess the contribution of responsible HRM of e-commerce business in increasing its revenues in the COVID-19 pandemic and crisis;
2. Study the international case study of responsible HRM in e-commerce during the COVID-19 pandemic and crisis and use it to define the outlines of a humanistic model of corporate social responsibility in e-commerce based on high-tech in an AI economy.

The discussion notes the contribution of the research to the literature. The conclusion formulates the key conclusions and substantiates the theoretical and practical significance of the results.

As a result of the research, the paper’s contribution to the literature is noted, the key conclusions are formulated, and the theoretical and practical significance of the results is described.

## Literature review

The theoretical basis of this research includes the concept of responsible HRM. The research object in this paper is the analysis of the responsibility of a business (on the example of e-commerce) toward its employees as a manifestation of corporate social responsibility. According to this concept, corporate social responsibility has two measurements. The first one is quantitative (Čater et al., 2023). It implies the improvement of the quantitative parameters of employment, the main of which are the creation of additional jobs and an increase in wages (Aparicio et al., 2023). These are formal parameters of HRM, which are subject to corporate accounting and, which are, as a rule, reflected in corporate reporting (Kumar et al., 2023).

The second measurement is qualitative; it is more complex, for it goes beyond the limits of the formal parameters of HRM. Here we speak of labor conditions—friendliness of personnel, unity of work teams, attentiveness and individual approach of the management to each employee, and comfort of workplaces (Chtioui et al., 2023; Rawshdeh et al., 2023). These conditions, in their totality, determine the opportunities for unlocking of human potential of the employees and their receiving satisfaction from labor; that is why, they have an important place in Maslow’s pyramid of needs (Vu, 2022).

Improvement of labor conditions, aimed at an increase in the quality of corporate social responsibility, is treated in this paper as humanization because it puts in the center of HRM each individual with their unique needs and implies the creation of favorable conditions for their satisfaction (Paruzel et al., 2023; Tuyen et al., 2023). This is the essential feature of the improvement in quantitative characteristics of corporate social responsibility, for in this case, employees are impersonal, and their needs are unified—which contradicts the idea of humanism (González-Ramos et al., 2023).

Ramos-González et al. (2022), Rawshdeh et al. (2019), and Zhao et al. (2021) studied in detail a quantitative criterion of corporate social responsibility, which is the creation of additional jobs. The essence of responsible HRM using this criterion is to supportыemployment and prevent the growth of poverty due to increased unemployment.

The period 2020–2021, for which official statistics have already been calculated and scientific studies are available, is notable not only for the peak of the COVID-19 pandemic and crisis but also for the height of the Fourth Industrial Revolution. A striking confirmation of this is the flowering of e-commerce as a field of business, which simultaneously achieved a high level of automation of economic activity and optimally met the requirements for social distancing (Dewalska-Opitek et al., 2022; Ding et al., 2022).

The transition from classic retail to e-commerce is accompanied by a reduction in the number of employees. The need for a large number of exhibition areas and sales halls is eliminated. Accordingly, employees are subject to reduction. The consequences of the described process of retail automation in the transition to the form of e-commerce are poorly understood and need to be studied in detail from the perspective of corporate social responsibility (Fedushko and Ustyianovych, 2022; Luo et al., 2022).

Based on the works of Chang et al. (2022), Music et al. (2022), Popkova (2022), Popkova and Sergi (2021, 2022), Rezapour et al. (2022), Romano et al. (2022), Sergi and Popkova (2022), which note the financial benefits of corporate social responsibility, this research hypothesizes (H_1_) that the humanization (qualitative criterion) of workplace organization provides more of an increase in e-commerce business revenue than an increase in the number (quantitative criterion) of jobs.

Im (2021), Parr (2022), and Schmidpeter and Winter-Ebmer (2021) present a negative interpretation of high technology from the perspective of corporate social responsibility because automation is seen as a path to technocracy in the workplace. Technocracy refers to an approach to the organization of workplaces that creates the most favorable conditions for using high technology; for people (workers), these conditions may be unfavorable. E-commerce is only possible due to automation and cannot be done without high technology. Therefore, in e-commerce, the risks of automation for responsible HRM are the highest and need to be studied separately.

Based on the works of Cinco (2021) and Gallego and Kurer (2022), which point out the benefits of high-tech for workplace organization, this research suggests hypothesis (H_2_) that the high-tech e-commerce model is preferable from the perspective of the humanization of corporate social responsibility (CSR) than the classical model (relying only on the Internet and mobile communications). It should be noted that prior studies investigating HRM and CSR in e-commerce companies mention the significance of HRM and CSR practices. However, these practices were studied in isolation, due to which the differences in their significance remain unclear.

Al-Shourbaji and Zogaan (2022) noted the critical importance of HRM for e-commerce and an increase in the effectiveness of companies that implement it and suggested distributing human resources in e-commerce with the use of a meta-heuristic algorithm and cloud technologies. Bai et al. (2021) also confirmed the importance of human resources and their management for the development of business in e-commerce. The authors offered to optimize the personnel resources of e-commerce companies in rural territories based on Big Data. Adam et al. (2020) proved that the development of human resources drives global e-commerce in the B2C market to a larger extent than access to ICT and the regulatory framework, confirming the main role of HRM in the development of companies в e-commerce.

In their turn, Zhang et al. (2023) noted the increased role and proved the anti-crisis effect of the corporate social responsibility practices, which were implemented by e-commerce platforms in China at the early stage of the COVID-19 pandemic. Wang and Yang (2021) proved that corporate social responsibility makes rural consumers change their attitude toward e-commerce and start using it actively, preferring it to offline purchases in the markets of dynamically developing countries.

Zhou (2021) elaborated on the significant contribution of corporate social responsibility to the growth of the effectiveness of cross-border e-commerce enterprises in China, demonstrating this contribution through the example of cross-border e-commerce enterprises based on the data envelopment analysis (DEA) model. Xiaolin et al. (2020) substantiated a significant positive (stimulating purchases) impact of corporate social responsibility on consumers’ purchase behavior in the E-commerce environment.

As a result of the literature review, we can conclude that the role of responsible HRM in the accrual of revenue by businesses is poorly understood and uncertain; the specifics of performing this role in e-commerce are unknown. To fill the identified gap, this research examines the experiences of e-commerce businesses that have demonstrated a high level of corporate social responsibility during the COVID-19 pandemic and crisis (2020–2021). Based on this experience, using e-commerce as an example, the authors clarify the causal relationship between business income from responsible HRM.

## Methods

To find an answer to the RQ and test both hypotheses, the authors choose the following concept of research organization (Table 1).

**Table 1:** The concept of research organization.

	Research objective		
Organization of research to solve the problem	To determine the contribution of responsible HRM of e-commerce business in increasing its revenues in the COVID-19 pandemic and crisis		To study international case studies of responsible HRM in e-commerce: the classical model vs. the high-tech model
Sample: research objects	Top 30 publicly traded e-commerce companies	The 16 most responsible companies in the retail industry in the USA	Leading global and Russian e-commerce business structures
Research period	2021	2020	2020–2021
Research method	Regression analysis, comparative analysis		Case study, comparative analysis
Condition of confirmation of the hypothesis	H_1_: A 1% increase in the quality of ESG management provides a greater increase in revenue than a 1% increase in the number of employees		H_2_: The high-tech model provides additional benefits from a humanization perspective

*Source:* Developed by the authors.

**Fig. 1:**
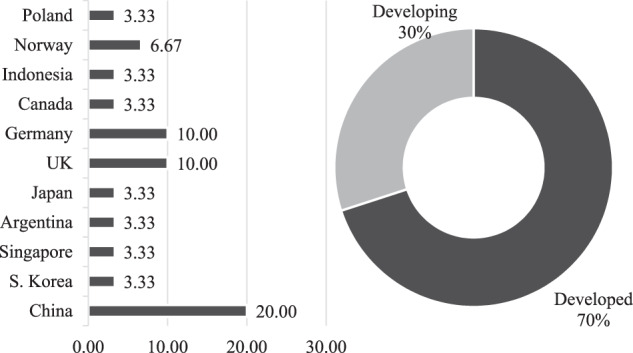
Geo-economic structure of the sample, %. *Source:* Calculated and constructed by the authors.

To solve the first problem of the research and determine the contribution of responsible HRM of e-commerce businesses in increasing revenues during the COVID-19 pandemic and crisis, the authors apply regression analysis. It is used for factor analysis of business income in e-commerce. Using the example of the top 30 publicly traded e-commerce companies in 2021, the authors conduct econometric modeling of the dependence of revenues of e-commerce business structures on the number of their employees. The geo-economic structure of the sample is shown in Fig. 1.

According to Fig. 1, the sample structure is dominated by developed countries (70%). However, there are also quite a few developing countries (Indonesia, Poland, Argentina, and China), the combined share of which is 30%. The sample includes 30 e-commerce companies leading in CompaniesMarketCap’s 2021 headcount rankings (2022). The empirical basis for this part of the research is shown in Table 2.

**Table 2:** The number of employees and revenues of the top 30 publicly traded e-commerce companies in 2021.

Rank	Name	Country	Employees	Earnings, bln USD
			emp	EN
1	Amazon	USA	1,523,000	13.36
2	Jingdong Mall	China	390	−0.65
3	Alibaba	China	2457	11.48
4	Meituan	China	100,033	−3.48
5	Coupang	S. Korea	68	−0.99
6	Sea (Garena)	Singapore	673	−2.05
7	MercadoLibre	Argentina	29,957	0.36
8	Rakuten	Japan	28,261	−1.58
9	Chewy	USA	213	−0.05504
10	Ocado	UK	19,347	−0.15
11	Zalando	Germany	17,199	0.1
12	Wayfair	USA	16,681	−0.98
13	Ozon	USA	14,834	−0.69
14	eBay	USA	108	−3.21
15	THG (The Hut Group)	UK	10,046	−0.19
16	Shopify	Canada	10	−2
17	Pinduoduo	China	9762	3.74
18	GoTo	Indonesia	9141	−1.21
19	Baozun	China	8821	−0.06391
20	Copart	USA	86	1.35
21	Adevinta	Norway	81	0.32
22	Vipshop	China	8013	0.89
23	Boohoo Group	UK	572	0.01043
24	Schibsted	Norway	5	−4.38
25	IAA-Insurance Auto Auctions	USA	45	0.38
26	Jumia	Germany	4484	−0.29
27	CEWE	Germany	4	0.07991
28	Groupon	USA	3675	−0.04882
29	Allegro.eu	Poland	3613	0.28
30	The RealReal	USA	3355	−0.22

*Source* Compiled by the authors based on CompaniesMarketCap (2022).

Using the example of 16 of the most responsible companies in the retail industry in the USA in 2020, the authors perform econometric modeling of the dependence of revenues of e-commerce business structures on the quality of ESG management. Company income statistics are taken from “Global 2000” for 2021 (Murphy and Contreras, 2022). ESG management quality statistics are taken for 2020 (year-end, i.e., relevant at the beginning of 2021). The empirical basis for this part of the research is shown in Table 3.

**Table 3:** ESG management and revenues of the most responsible companies in the retail industry in the USA in 2020.

Industry rank	Company	Score	Environmental Score	Social Score	Corporate Governance Score	Sales, $ bln.
			env	soc	gov	SL
1	Target	82.9	73.8	88.1	87.0	93.6
2	Home Depot	79.5	76.3	78.6	83.8	132.1
3	Best Buy	77.9	72.2	81.9	79.7	47.3
4	Lowe’s	77.5	62.0	88.9	81.6	89.6
6	Dick’s Sporting Goods	75.1	68.2	75.9	81.2	9.6
7	Gap	71.9	48.9	83.2	83.7	13.8
9	Kroger	70.6	61.7	69.4	80.9	132.5
11	Walmart	69.4	57.6	74.8	75.9	559.2
12	eBay	66.8	64.7	53.8	82.1	1.7
13	TJX	65.9	48.2	68.2	81.4	32.1
14	Sysco	65.0	46.3	68.1	80.8	45.9
16	Amazon	60.1	48.8	57.9	73.5	386.1

*Source:* Compiled by the authors based on Newsweek (2022) and Murphy and Contreras (2022).

Hypothesis H_1_ is considered proven if an increase in the quality of ESG management by 1% provides a greater increase in revenue than a one-percent increase in the number of employees. Two groups (Global top-30 and 16-US) were selected as observation objects in this paper because of the absence of unified statistics on the same indicators. A serious problem of studying modern international business is that, despite the universal principles (in particular, support of seventeen SDGs of the UN), there are no common standards of corporate reporting.

While in the sphere of financial accounting, this problem is solved by the International Financial Reporting Standards (IFRS), in the sphere of corporate accounting and reporting on sustainable development (including HR accounting, reporting on corporate social responsibility, and ESG reporting), there are no sectoral norms or standards at the least. Due to this, reporting on sustainable development seriously differentiates even among transnational corporations. This does not allow studying the number of employees and ESG practices by the example of the same sample of companies: because of the deficit of statistics.

Undoubtedly, it would be more reliable to use the same observation object, but this is impossible because of the absence of the necessary data for this research. The differences between various observation objects may potentially influence the experimental results, but only slightly, since all studied objects (in both samples) are large global companies, and US companies account for 30% in the sample of the Global top 30. That is why, we compare and analyze the Global top-30 and 16-US companies, which belong to the same sphere (e-commerce)—which makes their experience comparable.

For the second objective of this research, the authors studied the international case studies of responsible HRM in e-commerce in 2020–2021 using the examples of leading global (Amazon, Google, Wayfair, Alibaba, JD.com, eBay, and Walmart) and Russian (Lamoda, Wildberries, Yandex.Market, and Ozon) e-commerce business structures. A comparative analysis of the classical and high-tech e-commerce model from the perspective of humanization of corporate social responsibility is conducted. Hypothesis H_2_ is proven if the high-tech model provides additional benefits from the perspective of humanization.

## Results

### Contribution of responsible HRM of e-commerce business to increasing its revenues in the COVID-19 pandemic and crisis

As part of the first research task, the authors used regression analysis to determine the contribution of responsible HRM of e-commerce business to increase its revenues during the COVID-19 pandemic and crisis. The authors used the example of the top 30 publicly traded e-commerce companies in 2021 to determine the dependence of income of e-commerce business structures on the number of their employees. The results are shown in Table 4.

**Table 4:** The results of regression analysis for the top 30 publicly traded e-commerce companies in 2021.

Regression statistics						
Multiple *R*	0.6662					
*R*-squared	0.4438					
Normalized *R*-squared	0.4239					
Standard error	2.7502					
Observations	30					
**Variance analysis**						
	**df**	**SS**	**MS**	***F***	**Significance of** ***F***	
Regression	1	168.9632	168.9632	22.3387	5.85919*10^−5^	
Balance	28	211.7831	7.5637			
Total	29	380.7463				
**Regression coefficients**						
	**Coefficients**	**Standard error**	***t*-statistics**	***P*-value**	**Lower 95%**	**Upper 95%**
Y-crossing	−0.1903	0.5144	−0.3700	0.7141	−1.2440	0.8633
emp	8.7*10^−6^	1.8*10^−6^	4.7264	5.9*10^−5^	4.93965*10^−6^	1.2*10^−5^

*Source*: Calculated and compiled by the authors.

According to Table 4, the revenues of the top 30 publicly traded e-commerce companies in 2021 are explained by the number of their employees by 66.62% (multiple *R* = 0.6662; *R*^2^ = 0.4438). The econometric model takes the following form:

1 $${{{\mathrm{EN}}}} = - 0.1903 + (8.7 \ast 10^{ - 6}) \ast {{{\mathrm{emp}}}}$$

Based on model (1), If the number of employees increases by one person, revenues of the top 30 publicly traded e-commerce companies increase by 8.7*10^−6^ billion dollars. The average number of company employees in the sample is 60498. Using model (1), it is calculated that if the number of employees increases by 1% (60498*0.01 = 604.98 people), the revenues of the top 30 publicly traded e-commerce companies in 2021 will increase by $0.01 billion. To check the reliability of model (1), the authors performed an *F*-test. The significance of *F* was 5.85919*10^−5^. Consequently, model (1) corresponds to a significance level of 0.01. It has a tabular *F* of 7.64. The observed *F* is 22.3387; it exceeded the tabulated one; therefore, Fisher’s *F*-test is passed, and model (1) is reliable at a significance level of 0.01.

The example of the 16 most responsible companies in the retail industry in the USA in 2020 defines the dependence of revenues of e-commerce business structures on the quality of ESG management. The results are shown in Table 5.

**Table 5:** The results of regression analysis for the most responsible companies in the retail industry in the USA in 2020.

Regression statistics						
Multiple *R*	0.7617					
*R*-squared	0.5802					
Normalized *R*-squared	0.4228					
Standard error	129.7201					
Observations	12					
**Variance analysis**						
	**df**	**SS**	**MS**	***F***	**Significance of** ***F***	
Regression	3	186,082.4627	62,027.4876	3.6861	0.0622	
Balance	8	134,618.4798	16,827.3100			
Total	11	320,700.9425				
**Regression coefficients**						
	**Coefficients**	**Standard error**	**t-statistics**	***P*-value**	**Lower 95%**	**Upper 95%**
Y-crossing	3229.3766	936.3736	3.4488	0.0087	1070.0953	5388.6579
env	2.0167	4.1694	0.4837	0.6416	−7.5980	11.6315
soc	3.7272	4.1988	0.8877	0.4006	−5.9554	13.4097
gov	−43.2187	13.4359	−3.2167	0.0123	−74.2019	−12.2355

*Source*: Calculated and compiled by the authors.

According to Table 5, the revenue of the most responsible companies in the retail industry in the USA in 2020 is 76.17% (multiple *R* = 0.7617; *R*^2^ = 0.5802) explained by the quality of ESG management. The econometric model takes the following form:

2 $${{{\mathrm{SL}}}} = 3229.3766 + 2.0167 \ast {{{\mathrm{env}}}} + 3.7272 \ast {{{\mathrm{soc}}}} - 43.2187 \ast {{{\mathrm{gov}}}}$$

Based on model (2), if the environmental score increases by one point, the revenues of the most responsible companies in the retail industry in the USA will increase by $2.0167 billion in 2020. If the social score increases by one point, the revenues of the most responsible companies in the retail industry in the USA will increase by $3.7272 billion in 2020. The corporate governance score does not provide an increase in the revenues of the companies in the sample. To check the reliability of model (2), the authors conducted an *F*-test. The significance of *F* was 0.0622. Thus, model (2) corresponds to a significance level of 0.01. Tabular *F* is 2.92. The observed *F* is 3.6861. It exceeded the tabulated one, and Fisher’s *F*-test was passed. Model (2) is reliable at a significance level of 0.1.

Thus, a 1% increase in the quality of ESG management (social score) provides a greater increase in revenue for e-commerce businesses by $3.7272 billion, while a 1% increase in the number of employees provides only 0.01%. Consequently, hypothesis H_1_ is correct. This means that responsible HRM of e-commerce business makes a significant contribution to the increase in its revenues under the conditions of the COVID-19 pandemic and crisis. This means that the humanization of corporate social responsibility (improvement of labor conditions for the complete unlocking of human potential and increase in employees’ satisfaction with their labor) is preferable to the growth of formal quantitative characteristics of HRM (growth of the number of jobs).

It should be noted that a comparison of the results obtained from different observation objects (Global top-30 and 16-US companies) leads to certain errors. However, given the fact that all companies in both samples are large global companies and represent the same sphere (e-commerce), their experience is comparable. To specify the obtained quantitative results and reduce the mentioned error, caused by the difference in the samples, these results will be further supplemented by a quantitative—case—analysis, which covers the experience of various international companies from different countries.

It should be noted that the simultaneous improvement in the quantitative and qualitative characteristics of HRM in the process of manifestation of corporate social responsibility is complicated and cannot always be implemented in practice—in particular, in e-commerce. An increase in the number of personnel implies the cliché and mass character of HRM with the impersonality of employees. Contrary to this, to manifest the individual approach to each employee, it is often necessary to reduce personnel, for this allows decreasing the load on the management.

It should be also emphasized that the very essence of e-commerce involves automatization and reduction of personnel. That is why, an improvement in the quantitative characteristics of HRM, in particular, an increase in the number of jobs, is undesirable or even unattainable in e-commerce. A more preferable alternative is an increase in the quality of HRM, which stimulates humanization of corporate social responsibility—in this case, labor efficiency is improved, the quality of service is raised, customers’ loyalty grows, and the volume of sales and revenues of e-commerce companies takes place, while their expenditures for personnel remain unchanged (Vuong and Bui, 2023; Zhang et al., 2023). Thanks to this, the humanization of corporate social responsibility in e-commerce is characterized by high effectiveness not only from the social but also from the financial and economic position.

### International case studies of responsible HRM in E-commerce: the classical model vs. the high-tech model

To address the second research objective, the authors studied the international case studies of responsible HRM in e-commerce in 2020–2021 using examples of leading global and Russian e-commerce business structures. On its basis, the following outlines of the humanistic model of corporate social responsibility in e-commerce with the support of high technology in the AI economy are defined:

- Chatbots and voice assistants;
- Virtual try-on;
- Automatic classification of production;
- For future combinations, calculating the cost of goods, including logistics, taxes, and duties;
- Use of ready-made templates (registration forms);
- Mobile banking;
- Independent tracking of delivery by the consumer;
- Comments on the product on the Internet.

Table 6 provides a comparative analysis of the classical and high-tech e-commerce model from the perspective of humanizing corporate social responsibility (CSR).

**Table 6:** Comparative analysis of classical and high-tech e-commerce model from the perspective of humanization of CSR.

Business process of e-commerce	Classic e-commerce model	High-tech e-commerce model in an AI economy			
		Essence of the process	Technology	Benefits for CSR from a humanizing perspective	Case study
Advice on product selection	Call centers	Chatbots and voice assistants	AI	Reduced workload	Amazon Echo and Google Home
	Fitting rooms	Virtual fitting	VR/AR		Wayfair and Lamoda
Production planning and organization	Coordinated work of different departments	Automatic classification of production for future combinations, calculation of the cost of goods, including logistics, taxes, and duties	Internet of Things	Reduced complexity of work, Reduced responsibility	Google Tag Manager (GTM)
Acceptance (registration) of an order	Collecting data from the buyer	Use of ready-made templates (registration forms)	Cloud computing	Reduced workload and errors	Alibaba, Amazon, JD.com, eBay, Walmart
Acceptance of payment for orders, returns of goods	Bank card, terminal	Mobile banking	RFID technology	Reduced workload	Wildberries
Consultation on delivery time	Call centers	Independent tracking of delivery by the consumer	Geolocation	Reduced workload	Yandex.Dostavka
Gathering complaints and suggestions	Call centers	Online comments on the product	Blockchain	Ability to respond to requests at a convenient time	Ozon

*Source:* Developed by the authors.

As indicated in Table 6, the high-tech e-commerce model, first, provides a reduced workload for employees in providing advice on product selection. Call centers are replaced by chatbots and voice assistants based on artificial intelligence (AI). These are promising interaction mechanisms in the international market, making it possible to simplify and optimize marketing and advertising, logistics, customer service in foreign languages, and customs clearance. This is implemented in the e-commerce practices of Amazon Echo and Google Home.

Instead of fitting rooms, a virtual try-on is carried out. “Immersive” retail relies on interactive visualization technologies (virtual and augmented reality), which are particularly widely used in marketing communications to compensate for the lack of sensory information about a product. This is implemented in the practice of e-commerce of Wayfair and Lamoda (Delibaltova, 2021).

Second, the high-tech e-commerce model reduces the complexity of labor and reduces the responsibility of workers in the planning and organization of production. Coordinated work of different departments is replaced by automatic classification of production for future combinations and calculation of the cost of goods, including logistics, taxes, and duties. It is implemented in Google’s e-commerce practice, which has created the Google Tag Manager (GTM) marketing platform with the capabilities of transportation visualization needed to enable optimized e-commerce with the Internet of Things (IoT) (Kostin and Suboch, 2020).

Third, the high-tech e-commerce model reduces workload and errors in order intake (registration). The collection of data from customers is replaced by the use of ready-made templates (registration forms) using cloud computing (completed forms are stored in the cloud). The buyer once personal data and bank details once and selects the point of receipt of ordered goods, after which he or she can order goods “with one click.” This is implemented in the e-commerce practices of Alibaba, Amazon, JD.com, eBay, and Walmart.

Fourth, the high-tech e-commerce model reduces the burden on employees when accepting payment for orders and returning goods. Bank cards and terminals are replaced by mobile banking. This makes it possible to use high-tech payment mechanisms (mobile money and digital wallets, smart contracts). This practice is implemented by Wildberries through RFID technology: after verbal confirmation of the purchase of goods, money for their payment is automatically deducted from the consumer’s bank account.

Fifth, the high-tech e-commerce model reduces the burden on employees to advise consumers on the delivery timing of goods; call centers are replaced by self-tracking of delivery by consumers. This is implemented in the e-commerce practice of Yandex.Market. Geolocation allows the consumer to find at what stage (e.g., shipment or transportation) and in what geographical area (e.g., the logistics center of a particular region or city) the goods are at any time of delivery of ordered goods.

Sixth, employees can respond to calls at a convenient time when complaints and appeals are collected; call centers are replaced by consumers’ online comments on products. This is implemented in the practice of e-commerce of Ozon and virtually all marketplaces. Comments on the product serve as a signal to new customers. Using blockchain technology, vendor representatives handle appeals, thank customers for their purchases, offer to compensate them for losses, etc.

Thus, the high-tech model provides additional benefits for responsible HRM from a humanizing perspective. These benefits include a general reduction in the work and stress load and level of employee responsibility and a calmer and more predictable work schedule. Taken together, these benefits increase the attentiveness of e-commerce workers and their courtesy to consumers. They open up more opportunities for initiative, creativity, and talent. This reduces the risks of error (“human factor”) and increases productivity and the quality of services provided by e-commerce workers. That is, the volume of sales and, accordingly, the income of the e-commerce business increases due to the growth of employee involvement in the business and increasing their loyalty to the business.

## Discussion and conclusion

This research contributes to the literature by developing scientific provisions of the concept of responsible HRM. The research deeply explores the accumulated experience and reveals the causal links between the corporate social responsibility of business and its profits in the economic crisis caused by the COVID-19 pandemic. The authors systematized the experience of e-commerce businesses that have shown a high level of corporate social responsibility in the COVID-19 pandemic and crisis (2020–2021). This research clarified the role of responsible HRM in the accrual of revenue by e-commerce businesses. The new results are compared with the existing scientific literature in Table 7.

**Table 7:** Comparison of new results with existing scientific literature.

Comparable areas	Existing literature		New results obtained in the research that reflect the specifics of e-commerce
	Provisions	Sources	
The essence and criterion of corporate social responsibility	Creation of additional jobs: quantitative criterion of responsibility	Ramos-González et al. (2022), Rawshdeh et al. (2019), Zhao et al. (2021)	Humanization of the workplace: a qualitative criterion of responsibility
The nature of the impact of corporate social responsibility on business profitability in the COVID-19 pandemic and crisis	Increased customer loyalty: increased demand for responsible business products (improvement of external quantitative characteristics of business performance)	Santos et al. (2022), Asokan et al. (2022), Bianchet et al. (2021), El Khoury et al. (2022), Jasielska et al. (2022), Miller et al. (2022)	Improvement of employee loyalty: improvement of internal quality characteristics of employee labor and business operations
Interpreting high-tech and automation from the perspective of corporate social responsibility	Negative view of high-tech from a CSR perspective: automation as a path to workplace technocracy	Im (2021), Parr (2022), Schmidpeter and Winter-Ebmer (2021)	Positive CSR approach to high-tech: automation as a way to humanize the workplace

*Source*: Developed by the authors.

According to Table 7, in contrast to Ramos-González et al. (2022), Rawshdeh et al. (2019), and Zhao et al. (2021), it is advisable to use not only quantitative but also qualitative criteria for corporate social responsibility. The essence of this responsibility based on the qualitative criterion is not the creation of additional jobs but the humanization of jobs: the creation of a favorable environment for human resources of the business, comfortable for the disclosure of their human potential.

In contrast to Santos et al. (2022), Asokan et al. (2022), Bianchet et al. (2021), El Khoury et al. (2022), Jasielska et al. (2022), and Miller et al. (2022), it is indicated that corporate social responsibility increases business profitability in the COVID-19 pandemic and crisis not so much by increasing customer loyalty as by increasing employee loyalty. Improving the internal quality characteristics of workers and business operations contributes to the growth of revenues in e-commerce.

In contrast to Im (2021), Parr (2022), and Schmidpeter and Winter-Ebmer (2021), instead of a negative interpretation of high technology from the perspective of corporate social responsibility (automation as a path to technocracy in the workplace), it is more correct to interpret it positively: automation as a path to humanizing the workplace. The point of this humanization process is that, despite the reduction in the number of employees, it does not reduce but rather increases the level of corporate social responsibility of e-commerce businesses. The remaining workers in the jobs retained by them acquire more comfortable working and employment conditions.

The theoretical significance of the authors’ conclusions and results from the position of humanization of corporate social responsibility consists in their forming a scientific and evidence base for preference of humanization as the direction of responsible HRM compared to the alternative direction which is connected with the improvement of the quantitative characteristics of personnel management. This paper formed a systemic vision of the essence and advantages of humanization of corporate social responsibility from the social (growth of employees’ loyalty), financial and economic (growth of revenues of e-commerce companies), and technical (preference of the use of high technologies of the AI economy) points of view.

Thus, the research has answered the set RQ. The contribution of responsible HRM of e-commerce business to increasing its revenues in the COVID-19 pandemic and crisis is achieved by humanizing the workplace—improving working conditions (reducing the work and stress load and the level of responsibility, as well as providing a calmer and more predictable workday schedule). This increases the loyalty of the employee to the business (reduces the risk of errors, increases productivity and quality of service—attentiveness and courtesy). This increases the sales and revenues of e-commerce businesses.

It is proved that the humanization (qualitative criterion) of jobs provides an increase in revenues of e-commerce businesses more than an increase in the number (quantitative criterion) of jobs. Thus, an increase in the quality of ESG management (social score) by 1% provides a greater increase in revenue for e-commerce business structures by 3.7272 billion dollars, while the increase in the number of employees by 1% provides an increase of only 0.01% (hypothesis H_1_ is proven).

The high technology of the AI economy makes it possible to maximize the described contribution of responsible HRM e-commerce business in increasing its revenues. The authors developed a humanistic model of corporate social responsibility in e-commerce based on high tech in the AI economy.

In the humanistic model, advice on product selection is provided through chatbots, voice assistants, and virtual fitting. Production planning and organization involve the automatic classification of production for future combinations and the calculation of the cost of goods, including logistics, taxes, and duties. Acceptance (registration) of the order is carried out using ready-made templates (registration forms). Acceptance of payment for orders, as well as returns, is made through mobile banking. Advice on delivery dates involves self-tracking of delivery by the consumer. Complaints and wishes are collected through comments on the Internet.

The humanistic model of corporate social responsibility in e-commerce relies on the following high technologies of the AI economy: artificial intelligence (AI), virtual and augmented reality (VR/AR), the Internet of Things (IoT), cloud computing, RFID, geolocation, and blockchain. Numerous examples from international and Russian case studies have shown that the high-tech e-commerce model is preferable from the position of humanizing corporate social responsibility than the classical model (relying only on the Internet and mobile communications) (hypothesis H_2_ is proved).

The theoretical significance of the research results lies in the disclosure of the mechanism of increasing the income of e-commerce businesses using responsible HRM. The research proved the necessity of humanizing e-commerce jobs, revealed the essence of this process, and offered practical recommendations for it, relying on the high-tech AI economy.

The practical significance of this research is related to the fact that the developed humanistic model of CSR in e-commerce based on high technology in the AI economy will increase the profitability and, therefore, the resilience of businesses to future economic crises arising from pandemics.

Summing up the research, it should be noted that they are limited by the study of the experience of e-commerce, while in other spheres of the economy, it is necessary and, probably, preferable to focus on the humanization of corporate social responsibility, which, however, has its specific features and needs specific managerial approaches. Experience of other spheres is beyond the scope of this research, which is its limitation.

The evidence base on the example of e-commerce, which was formed in this paper, allows offering a hypothesis that on the whole in the economy (in all spheres), humanization of corporate social responsibility generates social (growth of employees’ loyalty) and financial and economic (growth of revenues of e-commerce companies) advantages, which are maximized during the use of high technologies in the AI economy. This hypothesis should be tested—through a thorough study of the humanization of corporate social responsibility in various spheres of the modern economy—in further studies in continuation of this research.

## Data Availability

All data used in this research was taken from the following public sources: 1. Newsweek (2022) *America’s most responsible companies 2020*. Available at: https://www.newsweek.com/americas-most-responsible-companies-2020/americas-most-responsible-companies-2020-retail. Accessed 7 Nov 2022. 2. CompaniesMarketCap (2022) *Top publicly traded e-commerce companies by number of employees* (as of November 7, 2022). Available at: https://companiesmarketcap.com/e-commerce/largest-e-commerce-companies-by-number-of-employees/. Accessed 7 Nov 2022. 3. Murphy A, and Contreras I (2022, May 12) Global 2000, 2022. *Forbes*. Available at: https://www.forbes.com/lists/global2000/?sh=bafc0225ac04 (Accessed November 7, 2022). 4. Delibaltova M (2021) E-commerce: current development challenges and ways to solve them by developing client relationship channels. *Kreativnaya ekonomika* [Creative Economy]. 15(5): 2063–2078. 10.18334/ce.15.5.112138. Available at: https://creativeconomy.ru/lib/112138. Accessed 7 Nov 2022. 5. Kostin KB, and Suboch AN (2020) Modern e-commerce business models. *Russian Journal of Innovation Economics*. 10(3): 1623–1642. 10.18334/vinec.10.3.110593. Available at: https://1economic.ru/lib/110593. Accessed 7 Nov 2022.
